# Pressure-Guided LSTM Modeling for Fermentation Quantification Prediction

**DOI:** 10.3390/s25175251

**Published:** 2025-08-23

**Authors:** Jooho Lee, Jieun Jeong, Sangoh Kim

**Affiliations:** Department of Food Engineering, Dankook University, 119 Dandae-ro, Dongnam-gu, Cheonan-si 31116, Chungcheongnam-do, Republic of Korea; jooho@dankook.ac.kr (J.L.); jjieun@dankook.ac.kr (J.J.)

**Keywords:** fermentation, artificial intelligence, internet of things, blockchain, LSTM

## Abstract

Despite significant advancements in sensor technologies, real-time monitoring and prediction of fermentation dynamics remain challenging due to the complexity and nonlinearity of environmental variables. This study presents an integrated framework that combines deep learning techniques with blockchain-enabled data logging to enhance the reliability and transparency of fermentation monitoring. A Long Short-Term Memory (LSTM)-based Fermentation Process Prediction Model (FPPM) was developed to predict Fermentation Percent (*FP*) and cumulative Fermentation Quantification (*FQ*) using multivariate time-series data obtained from modular sensor units (PBSU, GBSU, and FQSU). Fermentation conditions were systematically varied under controlled environments, and all data were securely transmitted to a Fermentation–Blockchain–Cloud System (FBCS) to ensure data integrity and traceability. The LSTM models trained on AAG1–3 datasets demonstrated high predictive accuracy, with coefficients of determination (R^2^) between 0.8547 and 0.9437, and the estimated *FQ* values showed strong concordance with actual measurements. These results underscore the feasibility of integrating AI-driven prediction models with decentralized data infrastructure for robust and scalable bioprocess control.

## 1. Introduction

Fermentation is one of the oldest biotechnological techniques, historically used to prolong the shelf life of foods and to improve their nutritional properties [1]. Its various forms, such as traditional, biomass-derived, and precision fermentation, are now integral to the development of novel food products and sustainable materials. Compared with conventional agricultural approaches, fermentation systems offer clear environmental benefits, including reduced land requirements, lower greenhouse gas emissions, and more efficient use of water resources [2]. The remarkable diversity of fermented foods across global cuisines can be largely attributed to the distinctive microbial communities involved in each process, especially bacteria, yeasts, and filamentous fungi [3]. By harnessing the enzymatic activity of these microorganisms, fermentation facilitates the decomposition of complex organic matter into simpler, more bioactive, and nutritionally enriched substances [4]. Yeasts, which are unicellular fungi prevalent in nature, have long been key players in fermentation technology. Once primarily associated with food preservation [5,6,7], fermentation is now appreciated for its contributions to flavor, texture, and functionality. While *Saccharomyces cerevisiae* remains the most utilized species, a variety of other yeasts, including *Saccharomyces exiguus* [8], *Candida milleri* [9], *Candida humilis* [10], *Candida krusei* (*Issatchenkia orientalis*) [11], *Pichia anomala* [12], *Pichia membranifaciens* [13], and *Yarrowia lipolytica* [14], are also widely employed depending on the specific fermentation context [15].

Artificial Intelligence (AI) has emerged as a core driver of the Fourth Industrial Revolution, influencing advancements across disciplines such as engineering, life sciences, healthcare, food technology, marketing, and finance [16]. This rapid evolution has been fueled by the integration of modern programming frameworks, the growing availability of large-scale data, and the enhanced accessibility of high-performance computing resources, all of which have facilitated the development of sophisticated machine learning models [17]. Machine learning and deep learning comprise a broad spectrum of algorithms and methodologies, particularly within the domain of supervised learning. Notable techniques include linear and logistic regression, decision trees, random forests, support vector machines (SVMs), and artificial neural networks (ANNs) [18]. Among these, recurrent neural networks (RNNs) have proven especially effective for processing temporal and sequential data, owing to their capacity to accommodate variable-length inputs. Enhanced architectures such as Long Short-Term Memory (LSTM) networks and gated recurrent units (GRUs) were designed to address the challenges associated with long-term dependency modeling and have found significant utility in fields such as probabilistic forecasting and medical image interpretation [19]. The convergence of AI and the Internet of Things (IoT) gave rise to the concept of the Artificial Intelligence of Things (AIoT) in 2017, which has since gained momentum as a transformative technology [20]. AIoT harnesses intelligent algorithms to extract value from sensor-generated data, enabling dynamic, context-aware systems. With advancements in sensor technologies, IoT platforms are now capable of continuously acquiring comprehensive environmental and behavioral data, thereby enhancing interactions between connected devices, users, and their surroundings [21].

Blockchain is a decentralized ledger technology that securely connects sequential blocks of verified transactions. Its foundational principle lies in the immutable storage of authenticated data, which has led to its widespread exploration and implementation across numerous sectors, particularly for enhancing data integrity and security in IoT environments [22,23]. By providing mechanisms for transparency, traceability, and verifiable provenance, blockchain strengthens trust in distributed networks. Through consensus protocols, it mitigates the inherent vulnerabilities in device-to-device communication, effectively addressing trust issues within IoT frameworks. The decentralized nature of blockchain ensures robust data integrity even in the presence of localized or node-specific attacks, and it enables the detection and isolation of malicious actors without degrading the performance of the overall system [24,25]. Moreover, blockchain architecture reduces operational overhead by distributing data management tasks, and it enhances the computational and storage efficiency of IoT systems by leveraging underutilized device resources. Additionally, it supports prolonged product life cycles by decentralizing maintenance processes and responsibilities across connected nodes within the IoT infrastructure [26].

## 2. Related Works

AIoT-based systems have been widely explored in diverse domains where the autonomous operation of products and services is required with minimal or no human intervention. Within such systems, AI serves as the cognitive engine, enabling the interpretation of contextual information, identification of patterns, and execution of data-driven decisions. Meanwhile, the IoT functions as the communication infrastructure, akin to a neural network, interconnecting devices to enable seamless data exchange and real-time responsiveness [27].

In a recent study, Lee et al. (2025) [28] introduced a Multi-Sensor IoT Device (MSID) integrated with a Blockchain-based Mobile IoT System (BMIS) designed to facilitate robust, real-time acquisition and immutable storage of environmental data. The proposed system demonstrated high operational reliability, achieving a 98.35% success rate in cloud-based data transmission and a 100% success rate in blockchain transaction execution. These results underscore the system’s potential for secure, long-term environmental monitoring with strong guarantees of data integrity.

Huang et al. (2023) [29] proposed a novel aquaculture production and distribution platform that integrates AIoT and blockchain technologies to restructure traditional seafood trading systems by removing centralized intermediaries. In this model, transactional data are securely encrypted and recorded within blockchain blocks, while AIoT mechanisms are employed to ensure the quality and traceability of aquatic products. This integrated approach facilitates decentralized, transparent, and secure peer-to-peer exchanges, thereby improving consumer confidence and streamlining operational processes across the aquaculture supply chain.

Zhang et al. (2023) [30] introduced a blockchain-supported secure Mobile Edge Computing (MEC) framework tailored for UAV-assisted AIoT systems, aiming to preserve data privacy during computation offloading processes. Their method involved formulating a joint optimization problem that simultaneously addressed latency and energy efficiency. By applying a block coordinate descent algorithm, the proposed solution achieved superior performance in minimizing delay and power consumption compared to conventional baseline approaches.

Chi et al. (2023) [31] proposed a trusted cloud–edge collaborative decision-making architecture for AIoT applications, designed to overcome key challenges including constrained edge computing resources, data privacy concerns, and secure device authentication. The architecture integrates lightweight Multi-Layer Perceptron (MLP) models for efficient local inference alongside blockchain technology to ensure trust and data integrity. Through simulations based on carbon emission datasets, the authors demonstrated the model’s practical viability and its effectiveness in enhancing both the security and intelligence of AIoT-enabled edge environments.

Hacinas et al. (2022) [32] applied a combination of AI and IoT technologies to improve the operational efficiency of biogas generation within anaerobic digestion systems. Their work highlights the potential of AIoT integration to optimize process control and enhance overall performance in bioenergy applications.

Rocha et al. (2024) [33] investigated the effects of different post-harvest and fermentation methods on the chemical and sensory properties of coffee, applying machine learning techniques to model and identify key compounds influencing flavor profiles. Their study demonstrated that specific fermentation conditions, such as SMF and SSF, significantly shaped acid production and enhanced cup quality through distinct sensory attributes.

Roell et al. (2022) [34] utilized machine learning approaches to model the dynamics of syngas fermentation by converting experimental observations into production rate-based data instances. Through this framework, carbon monoxide (CO) and hydrogen (H_2_) were identified as primary factors influencing alcohol yield. The study highlighted the effectiveness of algorithms such as random forests and SVM in capturing complex bioprocess behaviors, even when working with limited datasets.

Hosseinzadeh et al. (2022) [35] applied a range of machine learning algorithms to predict hydrogen production during the dark fermentation of wastewater. Among the tested models, random forest, SVM, Gradient Boosting Machines (GBMs), and AdaBoost demonstrated strong predictive performance. Using permutation-based variable importance analysis, the study identified critical metabolic parameters and trace elements as key determinants of H_2_ yield, offering valuable insights into the optimization of biohydrogen production processes.

Li et al. (2021) [36] proposed a hybrid modeling approach that integrates mechanistic models with LSTM neural networks to forecast pH levels and fermentation end-time in cheese production. This framework demonstrated high predictive accuracy, supporting enhanced process control and facilitating the implementation of digital twin technologies in dairy fermentation systems.

Du et al. (2022) [37] provided a comprehensive review of modeling strategies applied in industrial fermentation, encompassing mechanistic approaches such as kinetic modeling and Constraint-Based Modeling (CBM), data-driven machine learning techniques, and hybrid frameworks integrating both paradigms. The authors highlighted that the integration of machine learning with CBM and Computational Fluid Dynamics (CFD) offers enhanced capabilities for predicting process dynamics and facilitating bioreactor scale-up.

Zhu et al. (2020) [38] conducted a comprehensive review of soft-sensing techniques for fermentation process monitoring, with particular attention to data preprocessing, variable selection, and data-driven modeling approaches, including SVM, neural networks, deep learning, and fuzzy logic. The review further discussed optimization algorithms for parameter estimation, offering a valuable reference for the development of robust, real-time fermentation monitoring systems.

Li et al. (2021) [36] integrated mechanistic modeling with an LSTM network to predict pH and completion time in cream cheese fermentation. Outputs from the mechanistic model were used as inputs to the LSTM, yielding an R^2^ of 0.99 for pH prediction and an accuracy within 3 min for batches lasting 6–7 h.

Recent research has increasingly investigated the convergence of AIoT and blockchain technologies. The primary objectives of this research are to ensure the integrity and immutability of fermentation data through blockchain-based storage, to enable real-time and distributed computation via a decentralized infrastructure, and to evaluate the predictive performance of AI models in forecasting key fermentation outcomes based on environmental sensor data. As demonstrated by previous studies, encouraging results have been obtained in a variety of areas, including water quality surveillance, agricultural resource management, and energy optimization. However, the application of these results in the context of food fermentation remains limited. Few studies have simultaneously addressed real-time prediction accuracy and data integrity within a fully integrated AIoT–blockchain framework, highlighting a gap for solutions that effectively combine these capabilities to enable robust and scalable fermentation monitoring. This domain is characterized by significant process variability and intricate interdependencies among numerous parameters. This presents unique challenges that have yet to be fully addressed in the current literature.

In this study, an IoT-enabled monitoring platform was implemented with the goal to facilitate the continuous acquisition of environmental data under anaerobic fermentation conditions. To guarantee the integrity and security of the collected information, blockchain technology was integrated, ensuring tamper-proof data storage. The development of a deep learning model employing LSTM networks to predict critical fermentation indicators based on pressure and other environmental inputs was enabled by the utilization of this infrastructure. Potential application domains encompass beer and wine brewing, fermented dairy production, plant-based meat manufacturing, and bioethanol processing, where real-time monitoring plays a critical role in ensuring product quality and optimizing yield. This AI-centered framework underscores the transformative potential of integrating the IoT, blockchain, and deep learning in modernizing traditional fermentation practices. It also illustrates the viability of AIoT–blockchain convergence for advanced applications within the food industry.

Despite these global application prospects, the need for such innovation is particularly urgent in domestic Makgeolli production facilities, where fermentation is still often managed based on fixed time control rather than real-time process feedback. Seasonal fluctuations in ambient temperature frequently lead to premature termination of fermentation before completion or, conversely, over-fermentation when the optimal endpoint is missed. Premature termination results in wasted raw materials, while over-fermentation compromises the sensory quality and stability of the final product. Thus, the application of intelligent monitoring systems represents a critical step toward minimizing such inefficiencies and ensuring consistent product quality in traditional Korean fermentation industries.

## 3. Materials and Methods

### 3.1. Materials

Glucose (Hwami Glucose, Hwami Co., Ltd., Incheon, Republic of Korea) and dry yeast (Saf-Instant Gold, Fermentis Division of S.I. Lesaffre, Marcq-en-Baroeul, France) were used as the carbon source and fermenting agent, respectively. All solutions were prepared using ultrapure water (KSENC Ultrapure Water, KSENC Co., Ltd., Ansan, Republic of Korea).

### 3.2. Methods

For the preparation of fermentation mixtures intended for environmental data acquisition, ultrapure water was measured using an electronic scale (SW-1S; CAS Corporation, Yangju-si, Republic of Korea). Solutes, including glucose and dry yeast, were precisely weighed with a precision balance (GB303; Mettler Toledo Inc., Greifensee, Switzerland) and subsequently mixed.

Fermentation was carried out in a 10 L cylindrical vessel placed within a thermo-hygrostat chamber (LHT-2150C; Daihan Labtech Co., Ltd., Namyangju-si, Republic of Korea), with the temperature maintained at 37 °C in accordance with the procedure described by Masneuf-Pomarède et al. (2006) [39]. A constant amount of yeast (30 g) and distilled water (300 mL) was used across all experimental conditions. The composition of each fermentation mixture is presented in Table 1. The Data Collection Group (DCG1–4) varied the glucose concentration (5.71–13.16% *w*/*v*) to generate a range of fermentation profiles for training purposes. Conversely, the AI Application Group (AAG1–3) employed intermediate glucose concentrations (7.04–12.00% *w*/*v*) within the established range to simulate novel fermentation scenarios. These data were also incorporated into model training to improve prediction performance.

### 3.3. Collect Fermentation Data for LSTM

To develop a time-series dataset suitable for LSTM model training, three distinct sensor units were deployed to continuously monitor key parameters during the fermentation process. The system comprised: (1) a Pressure-Based Sensing Unit (PBSU), (2) a Gas-Based Sensing Unit (GBSU), and (3) a Fermentation Quantification Sensing Unit (FQSU). The FQSU integrates a vision system based on a Convolutional Neural Network (CNN) to evaluate fermentation progress through image-based pattern recognition. Detailed specifications of the sensors used for fermentation data collection are provided in Table 2.

#### 3.3.1. Sensor Units for Pressure and Gas Data Acquisition

(1) Pressure-Based Sensing Unit

**Figure 1:**
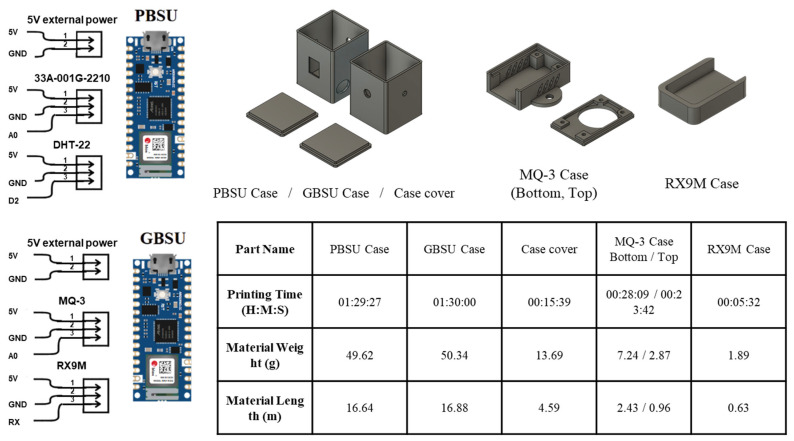
Electrical schematics and 3D-printed enclosures for sensor units.

Pressure-related fermentation data were collected using the PBSU, as shown in Figure 1. The PBSU consisted of a microcontroller unit (Arduino Nano 33 IoT; Arduino S.r.l., Monza, Italy) interfaced with two sensors: a 1 psi analog pressure sensor (33A-001G-2210; TE Connectivity, Plymouth, PA, USA) and a digital temperature–humidity sensor (DHT-22; Aosong Electronics Co., Ltd., Guangzhou, China). The sensor unit enclosure was fabricated using Hyper PLA_1.75 filament (Shenzhen Creality 3D Technology Co., Ltd., Shenzhen, China) and printed with a Creality Ender-3 V3 Plus 3D printer (Shenzhen Creality 3D Technology Co., Ltd., Shenzhen, China). STL files were processed with Creality Print slicer software (version 6.1.1) to generate the corresponding g-code for printing.

(2) Gas-Based Sensing Unit

Gas-related fermentation data were obtained using the GBSU, as shown in Figure 1. The GBSU was built with the same microcontroller unit used in the PBSU (Arduino Nano 33 IoT; Arduino, MA, USA). Two gas sensors were integrated with the microcontroller: an alcohol sensor (MQ-3, SEN040411; Henan Hanwei Electronics Co., Ltd., Zhengzhou, China) and a carbon dioxide sensor (RX-9M; Samyoung S&C, Incheon, Republic of Korea). All components were fabricated from the same materials and under the same printing conditions as the PBSU enclosure.

In the GBSU, raw analog values from the MQ-3 sensor were converted into cumulative gas exposure values using a cumulative increase algorithm. The cumulative gas concentration $C_{n}$ at time point n was calculated according to the following expression:

(1) $$C_{n}=\sum_{i=1}^{n}max(0,\;x_{i}-x_{i-1})$$

where $x_{i}$ denotes the analog output of the MQ-3 sensor at time point i, and $x_{i-1}$ is output at the previous time point.

#### 3.3.2. Fermentation–Blockchain–Cloud System

**Figure 2:**
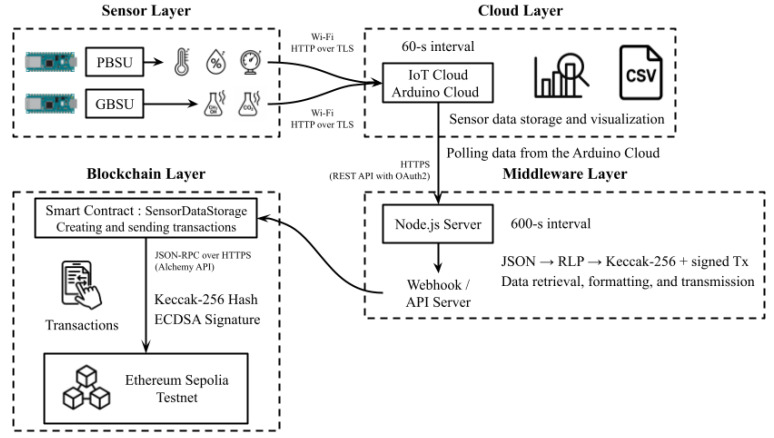
Overview of FBCS architecture.

The Fermentation–Blockchain–Cloud System (FBCS) was developed to enable real-time monitoring of fermentation data while ensuring immutability. As shown in Figure 2, the system architecture integrates cloud-based data visualization with decentralized blockchain storage to support transparent and verifiable fermentation tracking. Sensor readings from both the PBSU and GBSU were acquired at 60 s intervals and transmitted via Wi-Fi to the Arduino Cloud. These data were then periodically retrieved by a Node.js server, reformatted for blockchain compatibility, and uploaded to the Ethereum Sepolia Testnet.

A multi-layered FBCS was designed to ensure traceability and resistance to data tampering in fermentation monitoring. In the sensor layer, the PBSU and GBSU collected environmental parameters—including temperature, humidity, pressure, and gas concentrations—at 60 s intervals and transmitted them to the Cloud Layer via Wi-Fi using HTTP over TLS. The cloud layer employed Arduino Cloud services to store and visualize the data streams. In the middleware layer, a Node.js server periodically retrieved the cloud data, reformatted it into blockchain-compatible structures (JSON, RLP, Keccak-256), and generated ECDSA-signed transactions. These signed records were then transmitted to the blockchain layer, where they were immutably stored through interaction with a smart contract (SensorDataStorage) deployed on the Ethereum Sepolia Testnet via the Alchemy API.

To address potential communication instabilities, retry buffers and TLS-level error handling were implemented at the sensor–cloud interface, while API call retries with exponential backoff were employed at the cloud–middleware interface. Furthermore, transaction queues with nonce management and local caching were applied at the middleware–blockchain interface to ensure reliable data transmission and mitigate packet loss or temporary network failures.

#### 3.3.3. Consensus Algorithm and Security Protocols

To guarantee the integrity, availability, and traceability of fermentation monitoring data, the proposed system employs the Ethereum Sepolia testnet as its underlying blockchain infrastructure. Sepolia, a Proof-of-Stake (PoS) test network, replicates the consensus mechanism and security characteristics of the Ethereum mainnet, thereby providing a cost-effective and low-risk deployment environment [40]. The network operates using a GHOST-based consensus protocol integrated with PoS, in which validators are selected according to their staked ETH and reputation metrics [41]. This consensus mechanism significantly reduces energy consumption and shortens finality times, making it well suited for the periodic on-chain recording of fermentation data.

From a security perspective, Sepolia retains the cryptographic foundations of the Ethereum mainnet, including Keccak-256 hashing and the Elliptic Curve Digital Signature Algorithm (ECDSA). These mechanisms ensure that once sensor data are written to the blockchain via smart contracts, the records become immutable, independently verifiable, and fully traceable through distinct transaction hashes and associated block metadata.

#### 3.3.4. Smart Contract Deployment

To achieve tamper-resistant storage and ensure on-chain verifiability of fermentation data, a custom smart contract was implemented in Solidity (version 0.8.0) and deployed on the Ethereum Sepolia Testnet. The contract defines two distinct data structures for the PBSU and GBSU, which monitor temperature, humidity, pressure, and volatile alcohol and CO_2_ levels, respectively. The storeData() function appends incoming records to an on-chain array, while the getLatestData() function provides read-only access to the most recent entry.

The smart contract was compiled and deployed using Truffle (version 5.11.5), with transaction signing handled through HDWalletProvider connected to a MetaMask wallet. Sensor data, normalized in the cloud, were encoded via Web3.js, cryptographically signed using a locally stored private key, and transmitted to the blockchain through Alchemy’s HTTP endpoint to enable immutable data storage. Alchemy, a blockchain development platform, provides a comprehensive suite of tools and infrastructure services that streamline the development, monitoring, and scaling of decentralized applications (dApps) [42]. By incorporating industry-standard tools such as MetaMask and Alchemy, the system ensures secure wallet interactions and reliable access to on-chain sensor data [43].

### 3.4. Fermentation Quantification Sensing Unit

**Figure 3:**
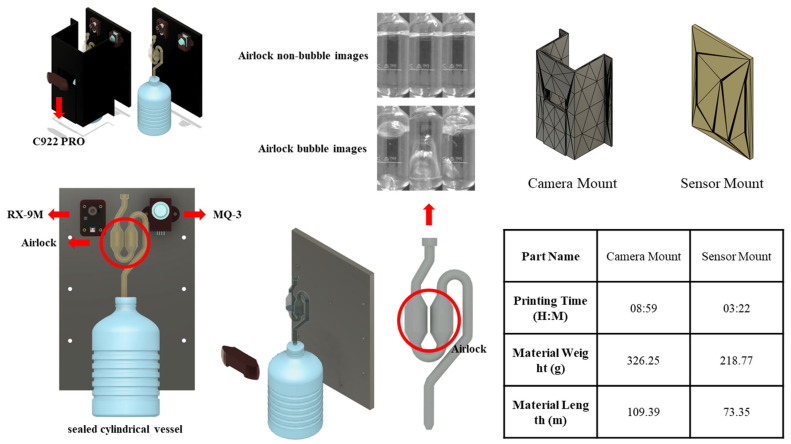
Chamber configuration of FQSU.

The Fermentation Quantification Sensing Unit (FQSU) was developed following the methodology of Jeong and Kim (2025) [44]. For model training, image data were acquired using a custom-designed fermentation chamber. Fermentation activity was indirectly assessed by monitoring CO_2_ release through an airlock system with a transparent U-trap filled with either distilled water or ethanol, enabling visual tracking of bubble formation. An HD C922 Pro Webcam (VU0060; Logitech Inc., Newark, CA, USA) was mounted inside the chamber on a 3D-printed holder, ensuring a stable and consistent viewing angle of the U-trap. To minimize ambient light interference, the chamber was enclosed, and image acquisition was performed continuously throughout fermentation. The FQSU configuration is illustrated in Figure 3.

#### 3.4.1. Image Data Collection for CNN-Based Fermentation Quantification Model

To generate image data for training the CNN-based Fermentation Quantification Model (CFQM), a standardized fermentation mixture was prepared by dissolving 20 g of glucose and 20 g of active dry yeast in 150 mL of distilled water and combining the solutions in a 1000 mL polypropylene vessel. To ensure an airtight environment and prevent gas leakage, all connections between the vessel, silicone tubing, and twin-bubble airlock were tightly sealed with Parafilm M (Bemis Co., Neenah, WI, USA).

Image acquisition was conducted under two controlled conditions: 37 °C using an electric food warmer (SAP-301; Ahpoongonyx Co., Hwaseong, Republic of Korea) and 33 °C at 50% relative humidity within a thermo-hygrostatic chamber (LHT-2150C). A Python-based script was used to capture 8-bit grayscale images (365 × 950 pixels) at 0.5 s intervals, with the camera field of view centered on the U-trap section of the airlock. The collected images were manually classified into two categories—those exhibiting visible CO_2_ bubble formation and those without—based on a ground-truthing procedure adapted from a previously reported method [45].

#### 3.4.2. Training Method for CFQM

Model training was carried out on a workstation (GF63 Thin 11UD, Micro-Star INT’L Co., Ltd., Taipei, Taiwan) operating under Windows 11 22H2, with Python (version 3.11.4) serving as the development environment. The acquired image dataset was randomly divided into training, validation, and test subsets at an 8:1:1 ratio using a Python-based directory-splitting script.

**Figure 4:**
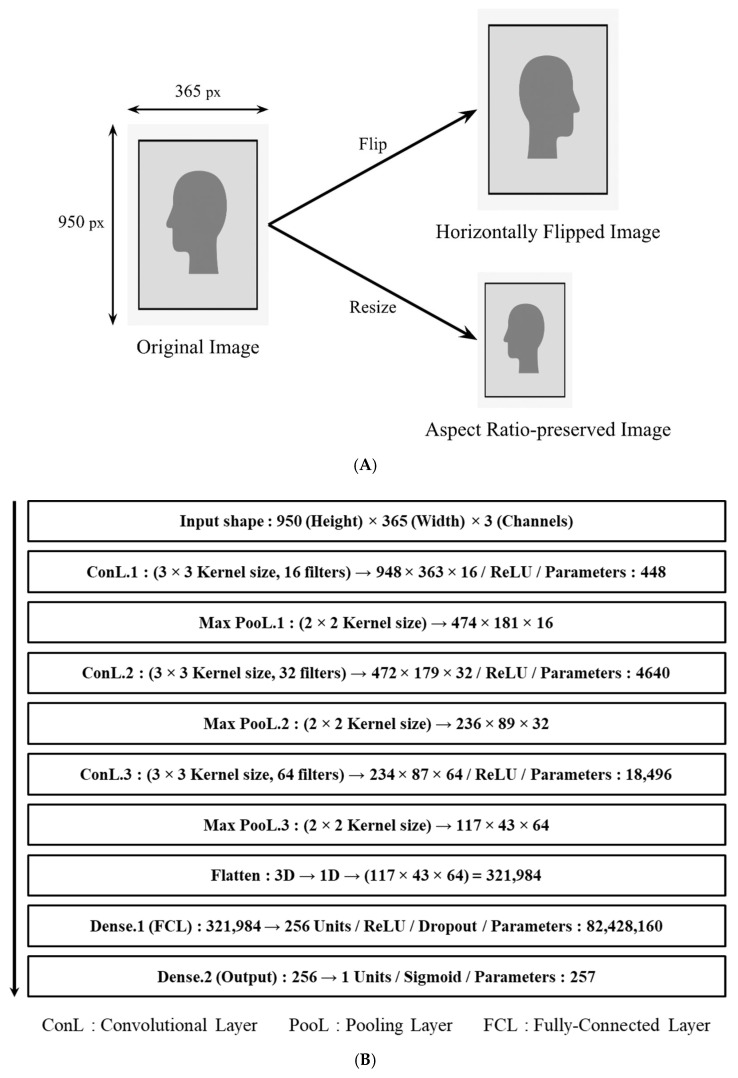
(**A**) Image augmentation strategy for dataset diversification. (**B**) Structure of CFQM.

The CFQM was implemented and trained using the TensorFlow library (version 2.17.0) in conjunction with Keras (version 3.4.1). To improve data diversity and reduce the risk of overfitting, image augmentation techniques including horizontal flipping and resizing with aspect ratio preservation were applied via Keras’ built-in image data generator, as depicted in Figure 4A. The dataset was processed in mini batches of 32 images, and model training was conducted over 12 epochs to assess classification accuracy and loss metrics across the training, validation, and test sets.

Model training was conducted on a workstation (GF63 Thin 11UD, Micro-Star INT’L Co., Ltd., Taipei, Taiwan) running Windows 11 22H2, with Python (version 3.11.4) serving as the development environment. The acquired image dataset was randomly partitioned into training, validation, and test subsets at an 8:1:1 ratio using a Python-based directory-splitting script.

The CFQM was implemented and trained using the TensorFlow library (version 2.17.0) together with Keras (version 3.4.1). To enhance data diversity and reduce the risk of overfitting, image augmentation techniques—specifically horizontal flipping and resizing with aspect ratio preservation—were applied through Keras’ built-in image data generator, as shown in Figure 4A. The dataset was processed in mini batches of 32 images, and model training was carried out over 12 epochs to evaluate classification accuracy and loss metrics across the training, validation, and test sets.

#### 3.4.3. Real-Time Fermentation Quantification Program

**Figure 5:**
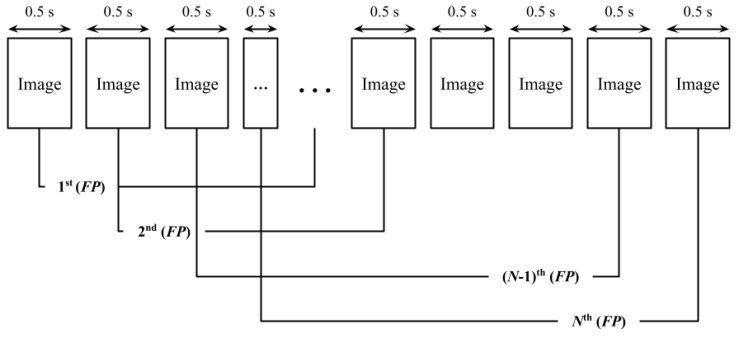
Structure of RFQP.

The Real-time Fermentation Quantification Program (RFQP) was developed to compute the Fermentation Percent (*FP*) from continuously captured image data. As illustrated in Figure 5, the RFQP divides each input image into 100 discrete segments and evaluates these regions to calculate the corresponding *FP* value. Implemented in Python, the program executes this process using the algorithm defined in Equation (2).

(2) $${FP}_{n}=\sum_{k=0}^{100}{FR}_{n+k}={FP}_{n-1}-{FR}_{n-1}+{FR}_{n+100}$$

The Fermentation Result (*FR*) indicates whether gas bubbles are present within a given image segment and is encoded as a binary value, with 1 signifying presence and 0 indicating absence. The *FP*, which reflects the extent of fermentation, is determined by counting the number of segments that exhibit gas bubbles within a predefined observation window. A formal mathematical definition of *FP* is provided in Equation (3).

(3) $$FP=\sum_{k=n}^{100+n}{FR}_{k}$$

Fermentation Quantification (*FQ*) represents the overall fermentation activity accumulated over time. It is formally defined as the cumulative sum of sequential *FP* values.

(4) $$FQ=\sum_{i=0}^{n}{FP}_{i}=\sum_{i=0}^{n}\sum_{j=n}^{100+n}{FR}_{j}$$

### 3.5. pH, Brix, and Alcohol by Volume

The initial and final pH values for each fermentation group were measured before and after the fermentation process using a calibrated pH meter (HI 11102; Hanna Instruments Inc., Woonsocket, RI, USA). The soluble solid content, expressed in degrees Brix (°Brix), was determined with a handheld digital refractometer (PAL-1; ATAGO Co., Ltd., Tokyo, Japan). All analytical measurements were conducted in triplicate to ensure reproducibility. Alcohol by Volume (ABV) was calculated according to the method described by Huang et al. (2024) [46]. The Original Gravity (OG) and Final Gravity (FG) were derived from the measured °Brix values using the following equation:

(5) $$Specific\;Gravity=1+\left(\frac{Brix}{258.6-\left(\frac{Brix}{258.2}\times 227.1\right)}\right)$$

The ABV was calculated concurrently from the OG and FG values, which were derived from the °Brix measurements, using the following equations:

(6) $$Alcohol\;By\;Volume\;\left(ABV,\;\%\right)=\left(OG-FG\right)\times 131.25$$

### 3.6. Data Preprocessing Methods

Data preprocessing and model training were performed on a Lenovo IdeaPad Gaming 3 15IHU6 (Windows 11 22H2) equipped with an Intel Core i5 3.11 GHz CPU and 32 GB RAM, utilizing Python (version 3.9.10) and PyTorch (version 1.13). A total of 21,332 fermentation data entries were collected from the PBSU, GBSU, and FQSU. Each entry contained six variables: fermentation time, humidity, temperature, pressure, MQ-3 gas concentration, and *FP*. Prior to training, all input features were normalized using the MinMaxScaler from the scikit-learn library. The normalization procedure is defined in Equation (7), and the corresponding inverse transformation for restoring the original data scale is shown in Equation (8).

(7) $$X_{std}=\frac{\left(X-X.\;min\right)}{\left(X.max-X.min\right)}$$

(8) $$X_{scaled}=X_{std}\times (X.\max-X.min)+X.min$$

MinMaxScaler normalization is particularly advantageous for high-dimensional datasets, as it preserves the relative relationships among feature values while mitigating distortions caused by differences in variable scales [47]. In this study, MinMaxScaler normalization was applied exclusively to the multivariate time-series data obtained from the PBSU and GBSU.

To format the time-series data for RNN modeling, a sliding window of five time steps was employed, generating input sequences from five consecutive observations to predict the *FP* value at the following time step. The resulting dataset was partitioned into training and validation subsets using an 80:20 split, with a fixed random seed (42) applied to ensure experimental reproducibility. The predictive model was constructed using an LSTM architecture consisting of two stacked layers with a hidden size of 64 units, followed by a fully connected output layer to capture temporal patterns in the input data. Training was conducted over 1000 epochs using the Adam optimizer with a learning rate of 0.001. The Mean Squared Error (MSE) loss function was adopted, and training was carried out in mini batches of 64 samples. These hyperparameter settings were chosen to achieve a balance between convergence speed and generalization capability.

The adoption of an RNN-based LSTM model was further motivated by prior observations that yeast sugar utilization can be reliably inferred from preceding fermentation product (*FP*) outcomes, making sequential modeling more suitable than static approaches. To this end, the binary classification of *FR* presence—distinguishing airlock bubbling from non-bubbling cases—ensured that the dataset remained tractable, while the simplicity of this dichotomy minimized risks of overfitting despite the limited sample size. Moreover, the *FQ* module acted as a noise filter, effectively dampening random fluctuations and alleviating potential overfitting. The LSTM parameters, including a hidden state size of 64 units, a dropout rate of 0.2, and early stopping based on validation loss, were selected to capture fermentation dynamics while maintaining generalization. Collectively, these design choices enabled robust *FP* prediction from sequential sensor data, while balancing accuracy, computational efficiency, and resilience to noise.

### 3.7. Fermentation Process Prediction Using Fermentation Process Prediction Model

To estimate *FP* values throughout the fermentation process, three distinct Fermentation Process Prediction Models (FPPMs) were constructed, each corresponding to AAG1, AAG2, and AAG3. These models were implemented using LSTM neural networks to effectively capture temporal patterns in the sequential environmental data. The model input consisted of five consecutive time steps comprising time, pressure, MQ3, humidity, and temperature variables, with the subsequent *FP* value serving as the prediction target. Prior to inference, input features were normalized using the MinMaxScaler fitted to the training data. Input sequences were generated using a sliding window method, converted into PyTorch tensors, and then processed by the pre-trained LSTM models. Each model architecture comprised two stacked LSTM layers (hidden size = 64) followed by a fully connected output layer. Model weights were restored using PyTorch’s load_state_dict() function, and inference was performed in evaluation mode to maintain consistency. Each LSTM cell employed PyTorch’s default activation functions, with tanh applied for cell state updates and sigmoid for gating mechanisms, while the fully connected output layer used linear activation. No dropout or batch normalization was incorporated, and all weights were initialized using PyTorch’s default Xavier uniform initialization scheme. Predicted *FP* values were inverse-transformed to recover their original scale. Model performance was evaluated using three standard regression metrics, Mean Absolute Error (MAE), Root Mean Square Error (RMSE), and Coefficient of Determination (R^2^), computed across the entire dataset.

### 3.8. Statistical Analysis

All experimental data are expressed as mean ± standard deviation, with each condition tested in triplicate (*n* = 3). Statistical analyses were performed in Python (version 3.10). Within-group comparisons were conducted using paired Student’s *t*-tests (scipy.stats), with significance defined at *p* < 0.05. For between-group comparisons of ABV, one-way ANOVA was applied, followed by Tukey’s Honest Significant Difference (HSD) post hoc test (statsmodels). Groups not sharing the same alphabetical letter were considered significantly different at the 0.05 level.

## 4. Results

### 4.1. Development of PBSU, GBSU, and FQSU

**Figure 6:**
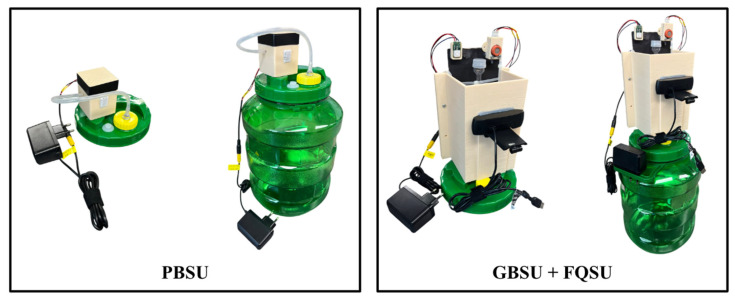
Configurations of sensor units.

To facilitate accurate fermentation monitoring, three modular sensor units were independently developed and structurally integrated with the fermentation vessels. Their finalized mechanical configurations are shown in Figure 6. The PBSU was designed to detect pressure variations during fermentation. A 10 L commercially available cylindrical PET tank served as the fermentation chamber, sealed with a valve-equipped lid. A silicone tube connected the valve to a pressure sensor housed in a custom 3D-printed enclosure, ensuring airtightness and accurate data collection. The GBSU, mounted on top of the FQSU airlock, was equipped with gas sensors to monitor fermentation gas emissions. The FQSU also employed a sealed PET tank connected to a twin-bubble airlock via silicone tubing to maintain internal pressure stability. To ensure reliable image acquisition for computer vision-based analysis, the area surrounding the airlock was darkened to minimize interference from ambient light. All three sensor units were assembled using commercially available, low-cost components suitable for laboratory-scale experimentation; however, their design and data acquisition principles allow for straightforward replacement with industrial-grade equivalents, enabling seamless adaptation for large-scale fermentation monitoring.

### 4.2. Sensor Data Acquisition Using PBSU and GBSU

**Figure 7:**
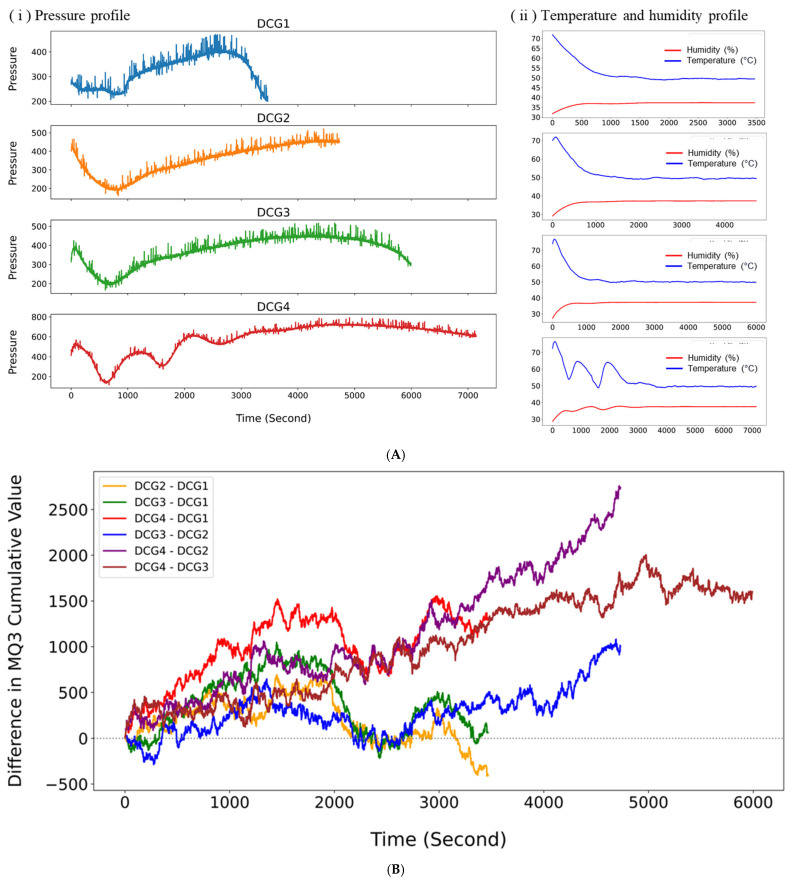
(**A**) Fermentation pressure and environmental conditions in DCG. (**B**) Comparison of cumulative MQ3 values among DCG.

Figure 7A illustrates the temporal progression of internal pressure in DCG as recorded by the PBSU during fermentation. The gradual increase in pressure reflects continuous microbial CO_2_ production, a metabolic byproduct of substrate degradation [48]. In DCG1, pressure rose slowly from an initial analog value of 279 to a peak near 470, followed by a sharp decline toward the end of monitoring, suggesting a relatively short fermentation phase likely due to early depletion of fermentable substrates. By contrast, DCG2 and DCG3 exhibited steadier, more sustained increases, reaching peak pressures of 521 and 516, respectively, and maintaining elevated levels for longer durations. These profiles indicate more stable and prolonged fermentation activity compared with DCG1. DCG4 showed the most pronounced pressure dynamics, beginning at 415 and steadily climbing to a maximum of 792. The recurring fluctuations observed in its pressure curve suggest multiple stages of microbial activity, potentially influenced by variable substrate availability or shifts in microbial community structure. Among all conditions, DCG4 appears to have undergone the most vigorous and sustained fermentation. Throughout all experiments, temperature and humidity remained stable.

Analysis of cumulative MQ3 sensor data collected from DCG1 through DCG4 using the GBSU revealed distinct variations in volatile gas production among the experimental groups. As shown in Figure 7B, DCG4 exhibited the highest cumulative increase, surpassing 2700 by the end of the measurement period, indicative of markedly elevated volatile compound generation. The cumulative differences between DCG4 and both DCG2 and DCG3 widened consistently over time, suggesting that the fermentation conditions in DCG4 promoted the most vigorous gas-producing activity. In contrast, the cumulative differences between DCG2 and DCG1, as well as DCG3 and DCG1, increased more gradually and displayed periodic fluctuations, reflecting comparatively lower gas output and fermentation efficiency under those respective conditions. These patterns align with previous reports indicating that enhanced microbial access to substrates, such as higher carbohydrate concentrations or inoculum levels, correlates with increased fermentation gas production [49].

### 4.3. Verification of FBCS Connectivity

The end-to-end integration test confirmed successful connectivity between the PBSU, GBSU, and the Arduino Cloud under controlled conditions. Although robustness measures were incorporated into the FBCS design, several potential communication errors remain possible across the multi-layered system. At the sensor–cloud interface, Wi-Fi instability could result in temporary packet loss, latency spikes, or jitter. While retry buffers and TLS-level error handling provide a degree of robustness, prolonged disconnections may still lead to missing segments of environmental data, which could limit the reliability of fermentation monitoring. To further enhance resilience, lightweight protocols such as MQTT with adjustable Quality of Service levels or redundant sensor gateways could be adopted in future implementations.

#### 4.3.1. Cloud-Based Visualization of Real-Time Sensor Data

**Figure 8:**
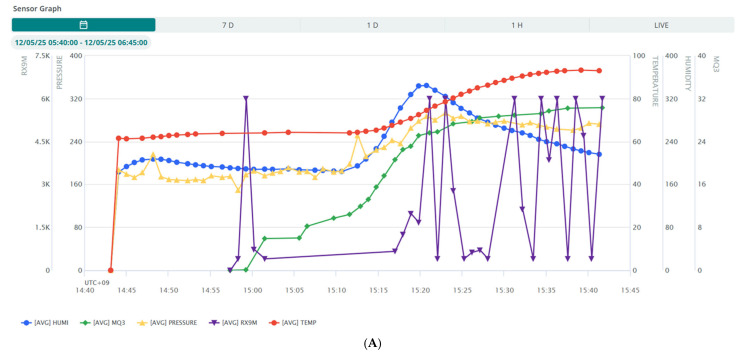

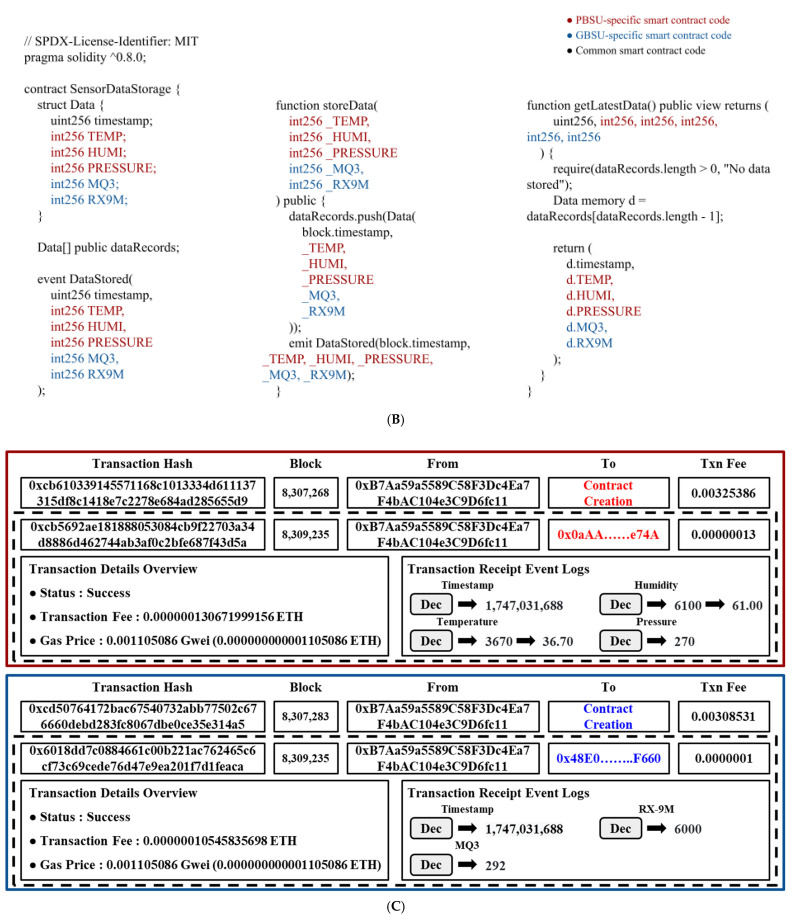
(**A**) Merged advanced chart displaying real-time sensor data on Arduino Cloud. (**B**) Smart contracts for FBCS. (**C**) Blockchain transactions and sensor data logs in FBCS.

To evaluate the real-time connectivity performance of the FBCS, sensor readings from both the PBSU and GBSU were transmitted to the Arduino Cloud at 60 s intervals using HTTP over TLS. As shown in Figure 8A, the combined time-series plot of five sensor variables demonstrates consistent and synchronized data transmission across the sensor and cloud layers. Among these variables, only the RX9M sensor data exhibited occasional transmission delays during specific intervals, suggesting transient latency in either data transfer or device response within the system architecture.

#### 4.3.2. Smart Contract Deployment and Functionality

To ensure tamper-resistant storage of fermentation monitoring data, the FBCS deployed two separate Ethereum-based smart contracts, individually assigned to the PBSU and GBSU. Each contract was designed to record time-stamped time-series sensor data on-chain in a structured format. As shown in Figure 8B, both contracts define custom data structures (struct Data) and dynamic arrays (Data[] public dataRecords) to sequentially log sensor outputs, with automatic timestamping enabled through block.timestamp. The PBSU contract records measurements of temperature, humidity, and pressure, whereas the GBSU contract logs gas sensor readings from MQ3 and RX9M. Both implementations incorporate a storeData() function to append new records to the blockchain and emit DataStored events as confirmation of successful transaction submission. Additionally, the getLatestData() function allows for retrieval of the most recent entry, enabling real-time access to current fermentation parameters.

Each contract was deployed via a dedicated migration script, with the entire process completed within 8 s without block delay, confirming stable transaction propagation. The PBSU smart contract was deployed at block number 8,307,268, with a final contract address of 0x0aAA87fA366e94d43562be3eb2B5aF1146f3e74A. The deployment consumed 325,386 gas units, corresponding to a transaction cost of ETH 0.00325386 at a gas price of 10 gwei. Similarly, the GBSU contract was deployed at block number 8,307,283, consuming 308,531 gas units at a total cost of ETH 0.00308531, and was assigned the address 0x48E0d848171d3A49e7a3DF9FfeC674D2146eF660.

While deployment overhead remained minimal, real-time operation involves continuous data submissions. In particular, the use of two smart contracts for PBSU and GBSU implies that multiple transactions may be triggered every 60 s when transmitting temperature, humidity, pressure, and gas sensor values. Prior evaluations of the Ethereum Sepolia testnet indicate median confirmation times of approximately 57 s, with smart contract interactions averaging 10–25 s under typical loads [50]. Concurrent transactions, however, can saturate 80–90% of block capacity, leading to increased delays of 15–30 s or more in high-throughput conditions [51].

Although the cost of execution on Sepolia itself is negligible, previous studies have shown that actual gas expenditure scales with contract complexity and storage overhead, with deployments of similar structured arrays consuming 3–4 × 10^5^ gas units per transaction [52]. These metrics suggest that frequent sensor transmissions (e.g., every minute across multiple nodes) may introduce performance bottlenecks in terms of both latency and scalability. To ensure scalability and robustness for industrial applications, strategies such as transaction batching or the adoption of Layer-2 solutions (e.g., rollups, sidechains) should be considered in future deployments.

#### 4.3.3. Validation of On-Chain Execution and Data Integrity

In this implementation, the blockchain layer was built on the Ethereum Sepolia testnet. Because all sensor data were first transmitted to a centralized Arduino Cloud service prior to blockchain storage, the system retains a potential single point of failure at the cloud layer. To evaluate the reliability and integrity of data transmission within the FBCS, actual blockchain transactions were examined using Etherscan, a widely used Ethereum blockchain explorer that provides transparent access to transaction records, blocks, and smart contracts [53]. Each transaction was retrieved using its unique transaction hash, and both metadata and emitted event logs were analyzed to verify successful execution and the accurate preservation of sensor data on-chain.

As shown in Figure 8C, the logged transactions include both contract deployment events and corresponding sensor data submissions. The Transaction Details Overview confirms the successful execution of all recorded transactions, including information on gas prices and transaction fees. Compared with traditional databases, this architecture entails higher write costs and latency; however, it provides transparent and tamper-evident records, ensuring that any post-recording modifications can be detected. This capability offers distinct advantages in applications requiring verifiable data provenance.

Taken together, this validation demonstrates that the FBCS effectively enables tamper-resistant and verifiable storage of sensor data. It ensures that fermentation parameters are not only captured in real time but also immutably recorded on the blockchain, thereby guaranteeing complete traceability throughout the monitoring process.

### 4.4. Development and Evaluation of CFQM

A total of 47,447 images were acquired using the FQSU housed within the LHT-2150C chamber. Among these, 42,752 images were classified as non-bubbling, whereas 4695 exhibited visible bubbling activity. The dataset was stratified and subsequently divided into training, validation, and test subsets to ensure balanced representation of both classes. Specifically, the training set comprised 34,201 non-bubbling and 3756 bubbling images, the validation set included 4275 non-bubbling and 469 bubbling images, and the test set consisted of 4276 non-bubbling and 470 bubbling images.

**Figure 9:**
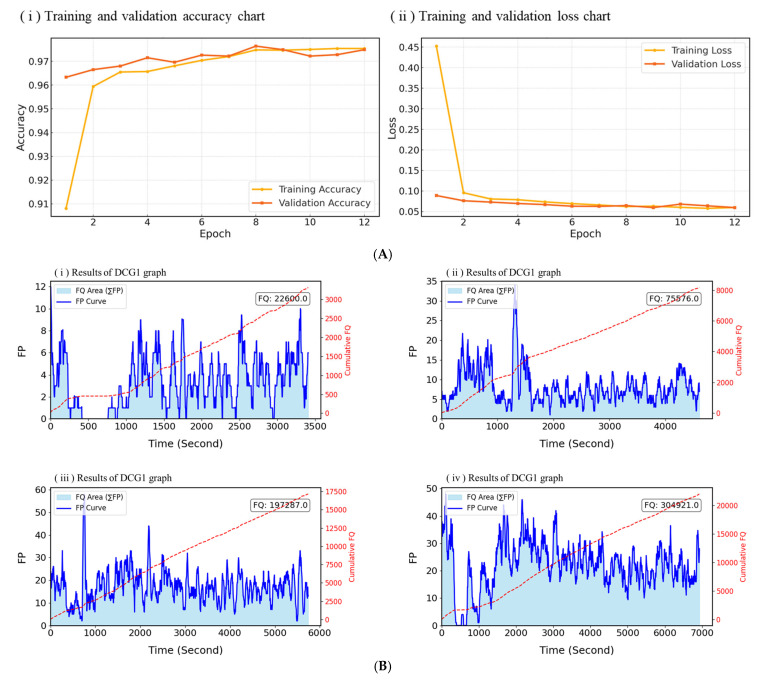
(**A**) Training and validation accuracy and loss of CFQM. (**B**) *FP* and cumulative *FQ* in DCG.

Figure 9A illustrates the CFQM’s performance during training and validation, presented in terms of accuracy and loss metrics. These results highlight the model’s ability to differentiate between bubbling and non-bubbling states under real fermentation conditions.

Figure 9B illustrates the *FP* trajectories together with the corresponding cumulative *FQ* values for four distinct fermentation trials. Here, *FP* denotes the instantaneous intensity of gas production, whereas *FQ* represents the temporal integration of *FP*, thereby serving as a quantitative indicator of the overall fermentation output.

In DCG1, *FP* values consistently remained below 12 throughout the monitoring period, leading to a comparatively low cumulative *FQ* of 22,600.0. By contrast, DCG2 exhibited an early surge in *FP*, surpassing 30, but the activity declined sharply thereafter, culminating in a final *FQ* of 75,576.0. DCG3 achieved the highest peak *FP*, reaching nearly 60, and sustained notable gas generation during the mid-phase, which contributed to a substantial cumulative *FQ* of 192,287.0. Finally, DCG4 displayed the most dynamic and prolonged gas production, characterized by pronounced fluctuations in *FP* values over an extended duration, ultimately yielding the highest overall *FQ* of 304,921.0.

### 4.5. pH, Brix, and Alcohol by Volume

**Figure 10:**
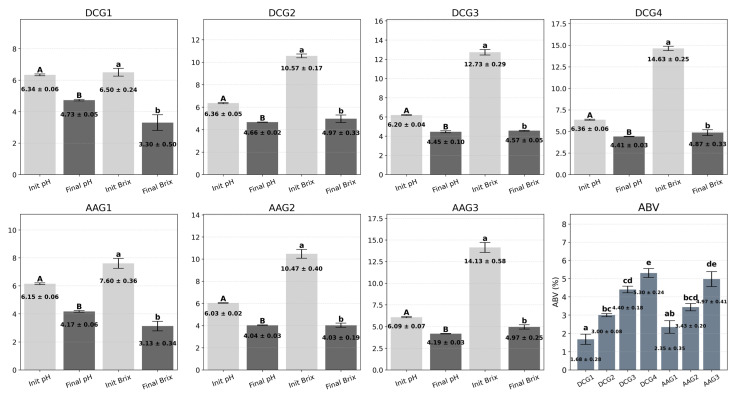
Analysis of pH, Brix, and ABV in DCG and AAG. Values are presented as mean ± standard deviation. Different uppercase or lowercase letters indicate significant differences at *p* < 0.05.

Figure 10 presents the initial and final pH and Brix values for each fermentation group, alongside their corresponding ABV percentages. Initial pH measurements ranged from 6.00 (AAG3) to 6.43 (DCG4), while final pH values declined significantly, falling between 4.01 (AAG2) and 4.77 (DCG1). Likewise, initial Brix values varied from 6.20 (DCG1) to 14.90 (DCG4). Following fermentation, residual sugar levels, as indicated by final Brix values, ranged from 2.80 (AAG1) to 5.40 (DCG2). All experimental groups demonstrated pronounced reductions in both pH and Brix, indicative of active microbial metabolism involving sugar utilization and acid production. These results are consistent with observations by Huang et al. (2024) [47], who reported that Saccharomyces cerevisiae efficiently metabolizes sugars while lowering pH as a byproduct of fermentation.

As shown in the ABV analysis, alcohol yields differed markedly across groups. DCG4 exhibited the highest ABV (5.65%), followed by AAG3 (5.44%) and DCG3 (4.64%), in alignment with their higher initial sugar concentrations and lower residual Brix values. Conversely, DCG1 produced the lowest ABV at 1.32%. Statistical analysis confirmed that these differences were highly significant (one-way ANOVA, F(6, 14) = 103.5, *p* < 0.0001, η^2^ = 0.978), with Tukey’s HSD post hoc test further indicating that groups not sharing the same alphabetical letter were significantly different at the 0.05 level.

### 4.6. Performance of FPPM

**Figure 11:**
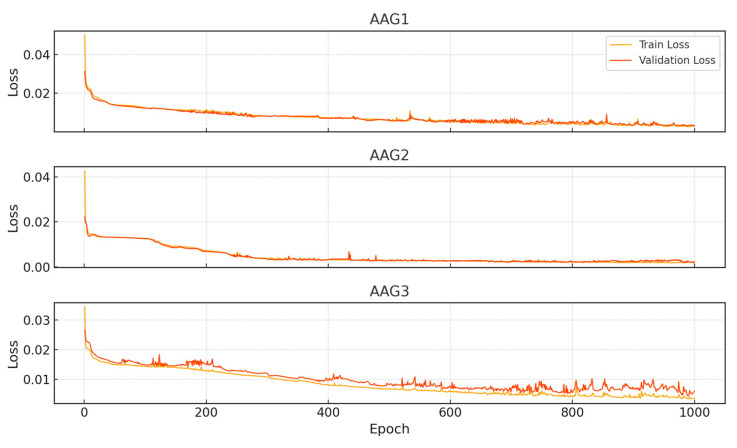
Training and validation loss of FPPM.

To assess the predictive performance of the FPPM, three separate LSTM models were trained using time-series sensor datasets obtained from the AAG1, AAG2, and AAG3 fermentation experiments. The number of training samples for each dataset was 25,324 for AAG1, 26,495 for AAG2, and 27,635 for AAG3. All models were trained for 1000 epochs under uniform training conditions. The AAG1 model achieved a final training loss of 0.00270 and a validation loss of 0.00314. For AAG2, the training and validation losses were 0.00181 and 0.00210, respectively. The AAG3 model exhibited a training loss of 0.00361 and a validation loss of 0.00596 (Figure 11).

### 4.7. Real-Time Prediction Using the FPPM

**Figure 12:**
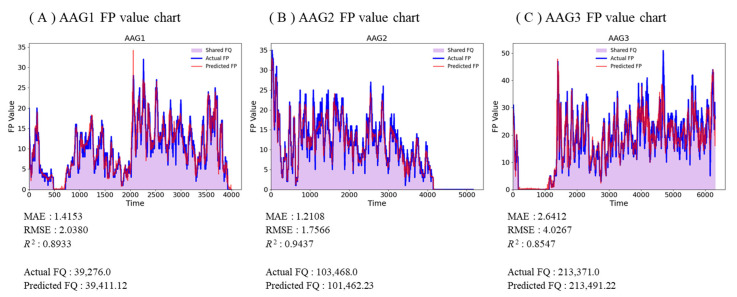
*FP* prediction results for AAG using FPPM.

To evaluate the real-time predictive performance of each FPPM, three independent fermentation scenarios were tested using their respective pre-trained LSTM models. Each model was applied to its corresponding dataset to generate predictions of *FP* values, which were then compared against actual measurements to assess predictive accuracy. Figure 12 presents time-series plots showing both the observed and predicted *FP* values for each fermentation condition.

For the AAG1 dataset, the model achieved a MAE of 1.4153 and an RMSE of 2.0380, with an R^2^ of 0.8933. The actual cumulative *FQ* was 39,276.0, while the predicted value was 39,411.12, indicating strong concordance between observed and predicted fermentation output. For AAG2, the model produced a MAE of 1.2108 and an RMSE of 1.7566, with an R^2^ of 0.9437. The corresponding actual and predicted *FQ* values were 103,468.0 and 101,462.23, respectively, demonstrating the model’s capacity to accurately capture fermentation gas production trends under this condition. In AAG3, the model yielded a higher MAE of 2.6412 and RMSE of 4.0267, with an R^2^ of 0.8547. Despite the increased dynamic variability in this dataset, the predicted *FQ* (213,491.22) closely aligned with the actual value (213,371.0), confirming that the model maintained reasonable predictive performance under more variable fermentation dynamics. Although high R^2^ values were achieved, predictive accuracy exceeding typical process variability may indicate partial overfitting. Nevertheless, the errors remained within a range acceptable for practical process control. In the context of fermentation, lower RMSE and MAE values indicate minimal deviation between predicted and observed *FP*, which under stable process conditions, is unlikely to affect cumulative *FQ* or final product quality.

## 5. Conclusions

This study proposed an integrated framework that combines sensor-based monitoring, blockchain-enabled data storage, and AI-driven analysis to support real-time management of fermentation processes. Custom-developed sensor units were effectively utilized to collect key variables, including pressure, gas concentration, and image-derived indicators of fermentation activity. The FBCS successfully ensured secure and traceable logging of these heterogeneous data streams, thereby providing both transparency and tamper resistance throughout the monitoring workflow.

The CNN-based CFQM demonstrated reliable classification of fermentation states by detecting bubble generation, while the real-time computation of *FP* and *FQ* provided a quantitative measure of fermentation intensity. Furthermore, the LSTM-based FPPM, trained on environmental time-series data, achieved strong predictive performance across AAG conditions, with R^2^ values consistently above 0.85 and minimal discrepancies between observed and predicted *FQ* values. These findings highlight the potential of sequential deep learning models for accurately capturing fermentation dynamics and supporting predictive process control.

Despite these promising results, several limitations must be acknowledged. The experiments were conducted under laboratory-scale conditions, where both the diversity and volume of data were inherently constrained. The overlap between the training and prediction datasets may have introduced bias, thereby limiting model generalizability. To overcome this, future work should emphasize large-scale, heterogeneous datasets that encompass a broader range of fermentation conditions, enabling the development of unified predictive models applicable to industrial-scale scenarios.

Moreover, real-world fermentation processes often involve greater variability and complexity than those observed under controlled laboratory settings. Addressing this challenge will require higher-sensitivity sensors, more diverse input modalities, and robust AI models capable of sustaining predictive accuracy under fluctuating operational conditions. In this regard, the proposed LSTM-based modeling framework holds considerable potential for real-time deployment, where it could enable dynamic monitoring, adaptive control, and optimization within intelligent fermentation systems.

The present study demonstrates the potential of an integrated fermentation monitoring system that leverages IoT-enabled sensors, cloud-based analytics, and blockchain-secured traceability to address the long-standing limitations of time-based control in industrial Makgeolli production. By enabling real-time detection of fermentation progress and dynamically adjusting process control, the system provides a reliable safeguard against premature termination or excessive fermentation. This innovation not only reduces raw material losses but also ensures consistent product quality, thereby enhancing consumer trust and industry competitiveness. Looking forward, the adoption of such intelligent systems may serve as a cornerstone for the digital transformation of traditional fermentation industries, paving the way for scalable, sustainable, and globally competitive production models. Ultimately, the incorporation of these technologies represents a decisive step toward the modernization of Korea’s cultural heritage beverages, ensuring both their authenticity and quality in future industrial applications.

## Figures and Tables

**Table 1:** Fermentation setup: component ratios and conditions.

Ingredient	Glucose (% *w*/*v*)	Yeast (% *w*/*v*)	Total Volume (mL)	Time (H:M)
DCG1	5.71	8.57	350	01:00
DCG2	8.33	8.33	360	01:20
DCG3	10.81	8.11	370	01:40
DCG4	13.16	7.89	380	02:00
AAG1	7.04	8.45	355	01:10
AAG2	9.59	8.22	365	01:30
AAG3	12.00	8.00	375	01:50

**Table 2:** Specifications of sensors for fermentation data acquisition.

Unit	Sensor	Measurement	Range	Interface	MCU/Processor
PBSU	33A-001G-2210	pressure	0–51.7 mmHg	Analog	Arduino Nano 33 IoT/SAMD21 Cortex^®^-M0+ (32-bit) (Seeed Studio, Nanshan District, China)
	DHT-22	T ^(1)^/H ^(2)^	−40–80 °C/0–100%	Digital	
GBSU	RX9M	CO_2_	400–6000 ppm	UART	
	MQ-3	alcohol	25–5000 ppm	Analog	
FQSU	VU0060	*FP* ^(3)^/*FQ* ^(4)^	1080p/30fps–720p/30fps	USB 2.0	–

^(1)^ T: temperature. ^(2)^ H: humidity. ^(3)^ *FP*: Fermentation Percent. ^(4)^ *FQ*: Fermentation Quantification.

## Data Availability

The data used to support the findings of this study can be made available by the corresponding author upon request.
